# The Role of Quorum Sensing in the Development of *Microcystis aeruginosa* Blooms: Gene Expression

**DOI:** 10.3390/microorganisms11020383

**Published:** 2023-02-02

**Authors:** Gisella Lamas-Samanamud, Armando Montante, Andrea Mertins, Duc Phan, Carla Loures, Fabiano Naves, Tony Reeves, Heather J. Shipley

**Affiliations:** 1 Department of Chemical and Materials Engineering, University of Kentucky, Paducah, KY 42002, USA; 2 School of Civil & Environmental Engineering, and Construction Management, University of San Antonio, San Antonio, TX 78249, USA; 3 USDA-ARS U.S Salinity Laboratory, University of California, Riverside, CA 92521, USA; 4 Department of Mechanical Engineering, Centro Federal de Educacao Tecnologica Celso Suckow da Fonseca, Angra dos Reis 20271-110, Brazil; 5 Department of Chemical Engineering, Universidade Federal de Sao Joao del Rei, Ouro Branco 36420-000, Brazil; 6 Int Med-Molecular Medicine, Wake Forest University School of Medicine, Winston-Salem, NC 27109, USA

**Keywords:** cyanotoxins, quorum sensing, microcystin, algal bloom

## Abstract

*Microcystis aeruginosa (M. aeruginosa)* is the dominant cyanobacterial species causing harmful algal blooms in water bodies worldwide. The blooms release potent toxins and pose severe public health hazards to water bodies, animals, and humans who are in contact with or consume this water. The interaction between *M. aeruginosa* and heterotrophic bacteria is thought to contribute to the development of the blooms. This study strives to provide a specific answer to whether quorum sensing is also a potential mechanism mediating the interaction of different strains/species and the expression by gene *luxS* or gene *mcyB* in *M. aeruginosa* growth. The *luxS* gene in *M. aeruginosa* PCC7806 is associated with quorum sensing and was tested by q-PCR throughout a 30-day growth period. The same was performed for the *mcyB* gene. Heterotrophic bacteria were collected from local water bodies: Cibolo Creek and Leon Creek in San Antonio, Texas. Results revealed that in algal bloom scenarios, there is a similar concentration of gene *luxS* that is expressed by the cyanobacteria. Gene *mcyB*, however, is not directly associated with algal blooms, but it is related to cyanotoxin production. Toxicity levels increased in experiments with multiple algal strains, and the HSL treatment was not effective at reducing microcystin levels.

## 1. Introduction

Harmful algal blooms (HABs) that result from the extravagant growth of cyanobacteria could produce cyanotoxins, lead to quality deterioration, and pose severe health risks to human consumption in both drinking and recreational waters [1,2,3]. Some of the most toxic substances to human liver are microcystins, the cyanotoxins produced by cyanobacteria such as *Microcystis aeruginosa* [4]. Consumption of microcystin-contaminated water could cause fatigue, vomiting, liver cancer, and even death. Direct contact with HABs such as from swimming could cause gastroenteritis, skin irritation, and allergic reactions. HABs also lead to severe environmental issues, such as deterioration in aquatic plants and fish. Recently, a few pet death cases were reported in association with a direct contact with toxins produced by green algae [5,6,7].

The United States Environmental Protection Agency (EPA)‘s investigation in 2007 showed that nearly 25.2% of the lakes in the U.S.A. have high cyanobacteria counts (above 20,000 cells/mL) and, thus, pose moderate to high risks regarding cyanotoxin exposure [8]. These numbers do not take into consideration algal blooms in seasonal creeks, as are common in places such as South Texas. *M. aeruginosa* is the dominant species causing HABs in freshwater lakes and low salinity estuarine and coastal ecosystems globally, such as Lake Erie, the Chesapeake Bay, and the in-land lakes of Florida in the USA; Lake Taihu in China; Lake Victoria in Africa; and the Baltic Sea in Europe [9]. One of the most severe events associated with record-high levels of microcystin concentrations in lakes occurred in Lake Erie [10,11].

Given that *M. aeruginosa* favors higher temperatures (above 25 °C), the frequency and scale of *M. aeruginosa* blooms are expected to rise with global temperatures [5]. Water contamination and warming conditions could potentially increase the frequency and the scale of events such as an increase in pet deaths associated with direct contact with toxins produced by cyanobacteria in several locations, especially during summertime, in which water bodies are used for recreational purposes.

Algal bloom is believed to be attributed to quorum sensing and the production of Homoserine Lactones (HSLs). Most studies involving HSL production attribute their presence to two set of genes, *luxI* and *luxR* [12,13], and their homologous genes, *luxA*, *luxB*, *luxC*, *luxD,* and *luxE* [14,15,16,17]. For instance, *luxI* is responsible for synthesizing the autoinducer, whereas *luxR* is responsible for responding to the autoinducer. If an organism does not have *luxI*, it can still respond to an autoinducer if *luxR* is present [17]. In algal blooms, *luxS* may be responsible for producing Autoinducer-2(AI-2), which is a signal molecule. This makes LuxS play the role of signaling for the activated methyl cycle. This mechanism drives the regulation of situational and environmental responses such as biofilm formation and changes in motility. LuxS is also believed to cause a chemical signal within the algae to begin the production and secretion of cyanotoxin. Hence, it is not autoinducer production that affects quorum sensing expression but whether the autoinducer is present, as well as the concentration in which it is found in the medium [18,19,20,21,22,23,24,25,26,27]

Toxin production is likely related to biofilm formation and social behaviors regulated by quorum sensing [28,29,30]. This cell–cell communication is regulated by the autoinducer homoserine lactones (HSL) in Gram-negative bacteria. Some studies have revealed that the biosynthesis of microcystins is given by different genes named *mcyA*, *B*, and *D*, among others [31,32,33,34,35,36,37].

This study focused on providing a specific answer to whether quorum sensing is a potential mechanism mediating the interaction of *M. aeruginosa* and heterotrophic bacteria and expressed by gene *luxS* or gene *mcyB* in *M. aeruginosa* growth, that is, to understand if the quorum sensing mechanism is responsible for both algal bloom and microcystin production.

## 2. Materials and Methods

### 2.1. Sample Collection

Samples were collected in sterile one-liter Nalgene bottles in the summer algal bloom of 2018 from surface water from Cibolo Creek (CC) and Leon Creek (LC) in San Antonio, Texas. Both samples were collected and characterized in terms of water quality parameters and filtered with 20 µm filters. As previously reported, the 20 µm mesh net retains most of the *Microcystis* colonies, which contain also the *Microcystis*-attached heterotrophic bacteria, whereas the <20 µm size fraction mainly contains free-living bacteria [38,39].

Two experimental designs were established in the laboratory setting: First, the filters were transferred to glass tubes with BG11 media (described in Section 2.2) to isolate the algal material and grown in the laboratory until they reached the same conditions of the algal sample already present in the lab, *Microcystis aeruginosa* PCC7806. Second, the filtrate was also transferred to glass tubes, as it was interesting to compare the algal bloom behavior of heterotrophic bacteria with the PCC7806 sample in the laboratory. In both systems, 23 mL of BG11 media was used.

Once the cultured environmental algal samples reached the same optical density (OD) values as *M. aeruginosa* PCC7806, the environmental algal samples were mixed in a 1:1 ratio with PCC7806 in BG11 media. In total, there were three glass tubes. One tube contained the PCC7806 by itself (2 mL of the strain in BG11 media). Another tube contained a mixture of 1 mL of PCC7806 with 1 mL of algal sample from CC, and the last tube contained a mixture of 1 mL of PCC7806 with 1 mL of algal sample from LC.

For the mixture of heterotrophic bacteria, algal samples were added in a 1:1 ratio and set up in duplicate (untreated and treated samples). The bacterial growth monitoring was conducted by measuring OD in a BioTek EPOCH microplate spectrophotometer at 600 nm. The room temperature was 20 °C. After the end of the experiment, samples were characterized in terms of water quality parameters following the standard methods for alkalinity, hardness, nitrate, nitrite, chlorine, phosphorous, and chemical oxygen demand (COD), as well as microcystin content, using a Beacon Microcystin Plate Kit. Samples were taken directly to the laboratory, and water quality and growth experiments were performed immediately to minimize variability.

### 2.2. Bacterial Growth

The bacterial strain *Microcystin aeruginosa* PC7806 isolated from Lake Erie was used as a standard for all experiments. The algae were grown in BG11 media, as mentioned in Section 2.1, which consisted of 1.5 g of sodium nitrate, 0.04 g dipotassium phosphate, 0.075 g magnesium sulfate heptahydrate, 0.036 g calcium chloride dihydrate, 0.006 g citric acid, 0.006 g ammonium iron (III) citrate, 0.001 g EDTA (disodium salt), 0.02 g sodium carbonate, 1.0 mL Trace Metal mix, and distilled water to a volume of 1 L. The method is based on the ATCC Medium 616 for Blue Green Algae. The Trace Metal Mix (1 L) was prepared, which consisted of 2.86 g of boric acid, 1.81 g manganese chloride tetrahydrate, 0.222 g zinc sulfate heptahydrate, 0.39 g sodium molybdate dihydrate, 0.079 g copper sulfate pentahydrate, and 0.0494 g cobalt (II) nitrate hexahydrate. The pH was adjusted to 7.1 with 1 M sodium hydroxide.

The preparation of the media and inoculation of algal samples consisted of inoculating 2 mL of *M. aeruginosa* PCC7806 in the stationary phase (optical density OD > 2) to a volume of 23 mL of BG11 media. *Microcystis* growth was monitored on a regular basis by the measurement of absorbance at 600 nm. Light exposure was kept constant for 12 h for 31 days.

Light was provided by a heat lamp with a 60 W bulb. All samples were grown on a VWR advanced digital shaker, which was set to 100 rpm. The incubation period for the samples was a total of 31 days with no addition of carbon source of media other than at the beginning of the experiment. Samples from the beginning and end of experiment were analyzed in terms of pH, conductivity, and phosphate levels according to standard methods. Samples of 2 mL were collected on a regular basis. Samples were taken on days 4, 5, 11, 15, 20, 26, 30, and 31. These samples were used for RNA extraction, reverse transcription, and q-PCR targeting the genes *16S rRNA* (*Eubacteria*)*, mcyB,* and *luxS*. A sample of 200 µL was also taken for microcystin test using ELISA kit for Microcystin.

### 2.3. The Interaction System of M. Aeruginosa and Other Algae from the San Antonio Area

The interaction system of *M. aeruginosa* and other algae from CC and LC were established in the laboratory. LC is a seasonal creek of mostly rainwater runoff. Because there is not a great volume of rain in the summer, the water bodies become hypereutrophic before drying, and this induces the toxin production related to HAB’s. CC is a permanent creek used for recreational purposes, but it also has a reduced volume during summer due to the lack of rain events.

After the filtration mentioned above, the filtrate was used for the second design of this experiment, and the filters were transferred to glass tubes with BG11 media to isolate the algal material.

### 2.4. The Interaction of M. aeruginosa and the Heterotrophic Bacteria

The experimental design consisting of the *M. aeruginosa* PCC 7806 strain from the axenic culture and the heterotrophic bacteria community isolated from the natural water served as a model system simulating the interaction of *M. aeruginosa* and bacteria in natural fresh water. Water samples from CC and LC were collected during the summer of 2018 during the algal bloom season.

The heterotrophic bacteria community resulting from the filtrate of the mucilage of *M. aeruginosa* colonies growing in these natural waters was used in this research. The filtrate was washed in sterile deionized water to dissolve the extracellular polymeric substance (EPS), and the colonies were disaggregated into single cells. The heterotrophic bacteria was separated from the single cells by filtration through filter paper of pore size 1.2 µm after ultrasonic treatment, which was used to kill the natural *M. aeruginosa* cells [39,40]. Then, the isolated heterotrophic bacteria (106 cells/ mL) were mixed with axenic *M. aeruginosa* PCC 7806 strain at a 1:1 ratio in the BG11 media. Previous laboratory studies demonstrated that heterotrophic bacteria are viable in such culture conditions and eventually cause *M. aeruginosa* to aggregate and form mucilaginous colonies [41].

A total of 3 glass tubes containing PCC7806 by itself (2 mL of the strain in BG11 media) were used. Another tube contained a mix of 1 mL of PCC7806 with 1 mL of heterotrophic bacteria from CC, and the last one contained a mix of 1 mL of PCC7806 with 1 mL of heterotrophic bacteria from LC.

The interaction experiment was conducted in duplicate, analogous to the system described in Section 2.3. The addition of HSL to the designated treated samples was performed on day 5 at a concentration of 50 mg/L of the HSL treatment. The duration of the experiment was 30 days with a constant light exposure of 12 h. Bacterial growth was monitored throughout the experiment, and samples were characterized in terms of water quality parameters and microcystin concentration at the end of the experiment.

### 2.5. RNA Extraction

The RNA extraction was accomplished using the All-Prep DNA/RNA mini kit by QIAGEN (Cat. No. 80204). The reverse transcription was accomplished by using the DNeasy PowerLyzer PowerSoil kit (Cat. No. 12855-100). SYBR Green Mastermix was used. Samples were placed into the iQ5 Multicolor Real Time PCR Detection System. The amplification reactions were performed under the conditions described in Table 1.

### 2.6. Bacterial Growth for 30 Days with and without Treatment

Another experiment was conducted simultaneously to the one described above with the introduction of a treatment with HSL (Sigma-Aldrich CDS006294). Because the HSL was added to the samples on day 4, treated samples were collected only on days 5, 11, 15, 20, 26, 30, and 31. All samples were grown under the same conditions as the ones described above.

### 2.7. Statistical Analysis

Samples were analyzed following standard methods for water quality parameters. pH was tested using a pH meter, AB15 plus Accumet Basic. Conductivity was measured with Seven Easy Metler Toledo. Phosphorous content was tested using Method 8190 and analyzed in an HACH DR/890 colorimeter. COD was tested using Method 8000, Digestor ECO 25 Thermoreactor, Velp Scientifica, and analyzed by DR2800 HACH Program 430 for low range.

### 2.8. Statistical Analysis

Significant differences were determined by using an unpaired one-sided Student’s t-test using Microsoft Excel. A *p*-value less than 0.05 indicates statistical difference. *p*-values lower than 0.01 indicate highly significant difference. Bacterial growth was performed in duplicates, whereas all sampling and data analysis were performed in triplicates.

## 3. Results and Discussion

This study strives to provide a specific answer to whether quorum sensing is also a potential mechanism mediating the interaction of different strains/species and the expression by gene *luxS* or gene *mcyB* in *M. aeruginosa* growth. The overall goal is to determine whether quorum sensing affects algal bloom and, if so, if it is influenced by either heterotrophic bacteria or a specific gene.

The rationale of taking samples periodically is to identify and compare gene expression with bacterial growth stages. *M. aeruginosa* PCC7806 growth was monitored, and results revealed that there are peaks on days 5, 15, and 20. After day 31, samples vary in growth, but peaks are not higher than the ones at the log phase. At this point and without any addition of media, samples are at the stationary or even the decay phase. This was observed by optical density absorbance of 600 nm. Overall, algal bloom is higher during the late log phase > 20 days.

Gene expression of *luxS* was also studied. Results revealed that this gene is present throughout the duration of the experiment with a well-defined peak on day 26. After day 31, some data were either too low to be determined, or they did not show any *luxS* expression. In fact, after day 30, no signal was detected.

The gene *mcyB* is likely associated with microcystin production as described by [37]. Results suggest that *mcyB* is commonly found at the early stages of bacterial growth, and it intensifies during the log phase. There is also a difference between samples collected early in the morning and those collected late in the afternoon. The higher the light exposition, the higher the gene expression. This shows how the time in which samples were collected influences the gene expression of *mcyB*. Based on the results from all three experiments, the following days were selected for sampling: 4, 5, 11, 15, 20, 26, 30, and 31, as described below.

There was a concern that the small volume used in these experiments would be affected by evaporation, and this must be considered. However, previous experiments conducted in a similar way [42,43] were also affected by the evaporation rate, which was not measured in this experiment. Future studies can consider different types of inoculation volumes and compare these results statistically to those found in this study.

### 3.1. The Interaction System of M. aeruginosa and Other Algae from the San Antonio Area

The average temperature of summer of 2018 was 83 °F (28.3 ℃) , and the average humidity was 68%, according to Weather by CustomWeather. Visually, algal samples from CC showed more ramifications and a dark green color, whereas samples from LC resembled PCC7806 samples in terms of appearance and color. Some studies indicated that instead of additional algal samples, heterotrophic bacteria naturally present in water bodies can favor algal bloom [44,45]. The isolation and sequencing of samples from CC and LC indicated Cyanobacteria for CC (not able to identify specific strain) and *Microcystis* for LC.

Samples grow during the early log phase and begin to decrease after the late log phase. Samples that were treated with HSL showed lower values for most samples, with the exceptions on days 20, 30, and 31, in which the *16S* was the highest. Once they start reaching the stationary phase, they begin to develop a biofilm on the top layer of the glass tubes, and most cells tend to precipitate at the bottom of the tubes. This generally happens at night when there is no exposure to light. For treated samples, the precipitation would be more frequent even during light exposure.

OD results showed that samples mixed with CC had more biofilm formation (twice as much as control), whereas samples with LC had the least bacterial growth (half of control). It is possible that samples with LC oversaturated or depleted the BG11 media, which could have been caused by a competition between both types of algal samples. If the behavior of all three samples is considered, algal samples from CC favored growth, whereas those from LC decreased growth.

For the treated samples, PCC7806 has a consistent bacterial growth, and both LC and CC samples maintain the same behavior of the PCC7806 strain. In fact, they even favor growth, although not to a statistically significant extent. The addition of HSL shifted bacterial growth, and samples with LC had the highest development. It is possible that HSL reduces or disrupts quorum sensing and, consequently, any inhibition that might happen if treatment is not added to it.

### 3.2. The Interaction of M. aeruginosa and the Heterotrophic Bacteria

Samples reached an OD value of 3, which was higher than the maximum of 2 from Section 3.1. Bacterial growth was favored by heterotrophic bacteria, even under treatment. Samples mixed with heterotrophic bacteria from CC behaved similarly to samples with only PCC7806. The only difference was at the early log phase, in which PCC7806 had a higher growth than the samples mixed with CC.

Samples mixed with heterotrophic bacteria from LC, however, had the highest bacterial growth. It is possible that samples from this creek had nutrients that favored the growth of PCC7806; or through symbiosis, these samples might have had heterotrophic bacteria that supported algal growth. If the optical density of all three samples is considered, heterotrophic bacteria from CC decreased growth, whereas those from LC increased growth. One possible explanation can be described by the nutrients present on each sample that were not lost by filtration. This result was the opposite of what was found in Section 3.1, in which CC algal samples favored the highest bacterial growth.

Treated samples showed a significant difference between bacterial growth from PCC7806 and the other samples. Treatment held the algal sample to a maximum OD of 1.5, whereas the other samples favored bacterial growth. One possible explanation is that the concentration of HSL was not enough to disrupt quorum sensing and prevent samples from growing, that is, if bacterial growth is related to quorum sensing. The treatment prevented samples mixed with LC from reaching an OD of 3.5, but they were still higher than those for PCC7806. Overall, treatment was not effective at reducing bacterial growth. In fact, it favored bacterial growth.

### 3.3. Gene Expression of M. aeruginosa PCC7806 and Control

*M. aeruginosa* PCC7806 was grown for 31 days, and samples were collected on days 4, 5, 11, 15, 20, 26, 30, and 31. Samples that received a synthetic HSL were named treated samples. Control samples without any treatment were referred to as non-treated. Treated and non-treated samples were performed simultaneously to minimize any error related to light exposition or any other systematic error. All collected samples were tested for Eubacteria *16S*, *luxS,* and *mcyB*.

In terms of the genes studied in this study, *luxS* tends to grow with time, whereas it decreases with time when HSL is added. Values are quite similar to each other, and they are not significantly different. This means that bacterial growth and *luxS* have a similar trend. It is not clear whether *luxS* causes bacterial growth or if the growth is influencing the gene expression. Statistical results reveal that both trends are similar. Treatment neither inhibited nor supported *M. aeruginosa* PCC7806 growth. The results revealed that any difference between both trends of treated and non-treated samples is not significant.

Treatment did influence *mcyB* but not to the extent that it is significantly different. The *p*-value of 0.05 was established as significantly different, and p-values < 0.01 were established as highly significantly different. When compared to bacterial growth and, consequently, *16S* and *luxS*, the results revealed that there is a significant difference between *16S* and *mcyB* (*p*-value = 0.04) as well as between *luxS* and mcyB (*p*-value = 0.01). Results also showed that the addition of treatment was highly significantly different when compared to *16S* and *luxS*. Both *p*-values were < 0.01.

**Figure 1:**
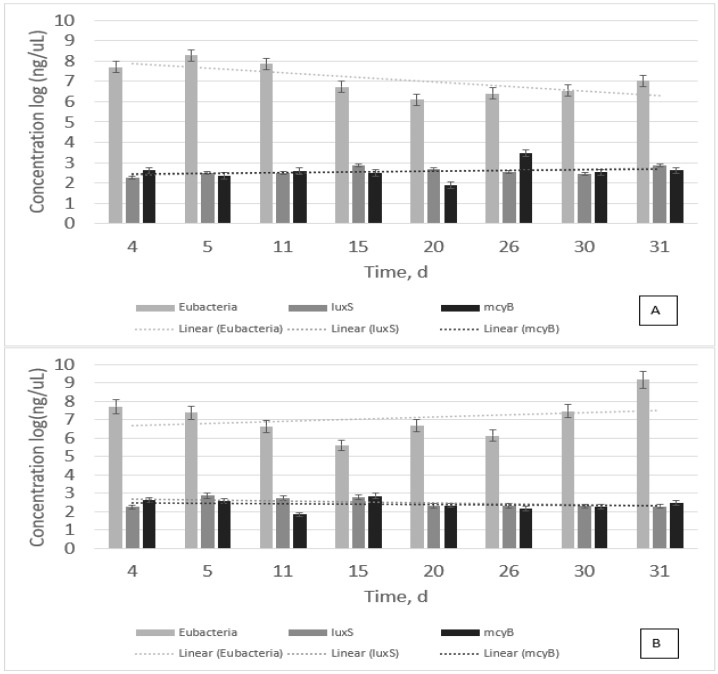
Combined results of gene expression for non-treated (**A**) and treated samples (**B**). Error bars represent the standard deviation of triplicates.

Figure 1 shows all the gene expressions tested for the samples. As biomass tends to decrease with time, *luxS* and *mcyB* tend to remain the same with time with a slight increase from 2 to 3 ng/µL (Figure 1A). Whereas biomass tends to increase with time (dotted line—trendline), *luxS* and *mcyB* tend to remain the same (Figure 1B). This result confirms that *luxS* is present, whereas *M. aeruginosa* is active and growing. It also infers that *luxS* is the quorum sensing signal that could possibly favor algal bloom. Results from Figure 1 also show that *mcyB* is present throughout the bacterial growth, and when HSL is added to samples, *mcyB* is reduced.

This study revealed that both *luxS* and *16S* are related to each other, that is, they are present during *M. aeruginosa* PCC7806 growth, and they have similar trends. However, *mcyB* did not show the same trend. In fact, statistical tests revealed that they are not related to each other. The question is whether microcystin production is related to *mcyB* or bacterial growth in general.

### 3.4. Comparison of All Genes to Microcystin Production

The ELISA test was used on all samples to determine the microcystin concentration during the bacterial growth period of 31 days. Because no microcystin was detected at the beginning of the experiment (when algae was diluted in BG11 media), results show microcystin content from day 4. However, a water characterization was conducted from day 0 (before media addition) and compared to that of day 31, as shown at the end of this section.

**Figure 2:**
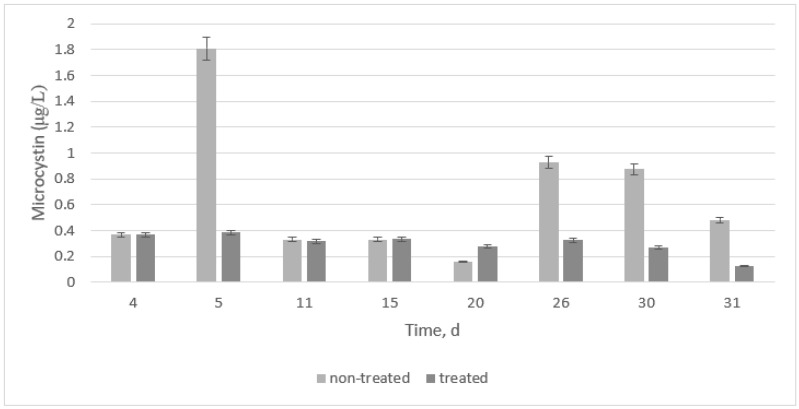
Microcystin production for non-treated and treated samples. Error bars represent triplicates.

Figure 2 shows two main things. First, the highest microcystin value was found on day 5 at the early log phase. This value increased again on days 26 and 30. Second, if treatment is added at the early log phase, microcystin values are kept at the lowest values.

Results were compared to data from each gene expression, and statistical results revealed that, when no treatment is added, *16S* and *luxS* are highly significantly different from microcystin production. However, *mcyB* is not highly significantly different from microcystin (*p*-value > 0.01). The peak on day 5 is most likely an outlier if the t-test analysis skewed the data. When all results were compared to the treatment, a highly significant difference was found for all cases. The treatment was indeed efficient for this type of *M. aeruginosa* if it was started at the early log phase.

The gene *mcyB* is not the only gene associated with microcystin production. In fact, several studies have determined the importance of these genes in toxin production [31,32,33,34,35,36,37]. A preliminary study [42] revealed that even if you have a depletion of other genes, there is still a chance of finding microcystin. However, when there is the depletion of *mcyB*, there is little or no microcystin production. Treatment with HSL added in this research showed a similar result to that found with the depletion of the gene, showing the importance of quorum sensing in determining the cause of algal blooms and toxin production, as well as preventing the toxin from being released or produced in the water body.

### 3.5. Water Quality Parameters and Analysis

Microcystin values from Table 2 are at least 1.5 times higher than values found at the early stage and freshwater samples (0.5 µg/L). All the values (untreated and treated) are higher than the maximum value of microcystin in water bodies recommended by the World Health Organization (WHO)—higher than 1 µg/L except for LC interaction for the untreated samples. A possible explanation for this result is that the HSL used was not efficient in preventing toxicity, as can be seen by the results for PCC7806 by itself. These results also revealed that the interaction of multiple algae tend to maintain an average value. This could indicate that one predominant organism overgrew the others, or that they are similar types of algae, and growth is limited by the available nutrients in the system.

There are two ways of knowing that an algal bloom episode is happening. First, the pH is more alkaline. If the pH can be monitored and controlled at neutral, it should prevent algal bloom from developing. Second, COD levels are risen. If there is an increase in the organic matter, it is consequently associated with bacterial growth. Nonetheless, these tests are not entirely necessary because algal blooms have a strong capability of turning water bodies green, which is visible to the human eye.

#### 3.5.1. Water Quality Analysis of *M. aeruginosa* and Other Algae from the San Antonio Area

Other measured parameters included pH, conductivity, phosphorous, and COD. The pH for all samples rose to above 9. Conductivity was reduced for all samples. Samples were retested, and they still displayed the same values. No additional media was added to the samples until the end of the experiment. There might be the possibility of an interaction with some of the components of the media.

The concentration of phosphorous is essential in the development of algal blooms. The higher number seen at the end of the experiment is due to the high concentration of phosphorous added to the BG11 media at the beginning of the experiment. The value of 0.5 is of the algae without media. The value of phosphorous increased considerably for all samples, but the HSL treatment maintained these values lower than those of untreated samples. This suggests that the treatment favored the consumption of phosphorous from the media.

The 30-day COD values were at least three times higher than the initial samples for all the experiments. Treated samples showed a lower COD in comparison with untreated samples. This could be an indicator that treatment can prevent some biomass formation, at least for the interaction systems. No difference from untreated and treated COD values was found for PCC7806. All these samples would require some type of treatment to be consumed by the population because values are higher than recommended values.

Although hardness remains the same for all samples, the alkalinity was reduced for both untreated and treated samples. Chlorine showed higher values than the initial samples. The maximum chlorine concentration was found for PCC7806 for treated samples in which the value was 3 mg/L. It is unclear why this value would be so high, because the concentration of chlorine in the media is about 10% of the result found. Nitrate and nitrite increased for untreated and treated samples for PCC7806 and treated samples of the interaction system with CC. The 30-day duration of the experiment could have favored the nitrification process at that stage, and this influenced the values found for these samples.

At the 30-day point, and if compared to Figure 1 and Figure 2, samples were already decaying. Overall, some major water quality parameters increased after 30 days, and the treatment was relatively effective in preventing quorum sensing and biomass formation. However, the treatment used was not effective in preventing microcystin production. Perhaps another type of HSL would be more effective, or the dosage could be corrected to prevent microcystin production.

#### 3.5.2. Water Quality Analysis of the Interaction of *M. aeruginosa* and the Heterotrophic Bacteria Study

Microcystin values for all samples grew after 30 days, with PCC7806 levels becoming three times higher. HSL treatment did not have any influence on the algal sample; however, it reduced microcystin levels when compared to untreated samples. Microcystin levels at the end of the experiment were higher than the values recommended by the WHO. However, these values were lower than the first setting of these experiments. Treatment was not effective in reducing bacterial growth; it considerably maintained microcystin levels within the recommended limits. A possible explanation is that the treatment does not influence microbial growth, but it influences toxin production. Thus, algal bloom and toxin production are not necessarily dependent on one another.

All samples were basic after 30 days, and conductivity decreased for PCC7806 and remained relatively constant for samples mixed with CC and LC. Nitrate increased in the interaction experiment with treatment. This is common in water bodies when organic material begins to decompose and is also an indicator of high nutrient level and lower oxygen. In terms of COD, there is a reduction of COD for mixed samples, whereas it increased for untreated PCC7806. Phosphorous content increased with treatment and could have been related to the increase in nitrate as well.

The most significant result from Table 2 is the reduction of microcystin content. This situation would be ideal for maintaining toxin levels within the recommended values. Therefore, this HSL could potentially be used to treat algal blooms under specific conditions. Although results showed unwanted toxicity or proliferation of microcystin, there are control measures and possible applications of recovered biomass as described in [46]. Joniver et al., 2021 [47] discuss the opportunity to create economically viable products from the algal biomass; this would be a profitable approach and an alternative to the negative environmental and economic effects on water bodies, which also include energy production [3,48].

#### 3.5.3. Comparison between Both Settings

It was interesting to compare both settings to predict how algal samples would react to an environment with either one of the conditions. This could possibly suggest how algal samples would react to real-life scenarios in which they are commensal with other types of algae and most likely other heterotrophic bacteria.

Untreated samples were not stable for a mix of algal material, as described in Section 3.1, in which maximum OD values were 2. Untreated samples with heterotrophic bacteria, however, were more stable, and they grew to an OD of 3.5. This suggests that algal bloom and bacterial growth are more related to symbiosis with heterotrophic bacteria, which consequently favors the bacterial growth. The answer to “What causes algal bloom?” can be related to the concentration and interaction of heterotrophic bacteria in the medium.

Treated samples for a mixture of organisms changed the behavior of multiple algae species to be more similar. In the case of heterotrophic bacteria, it did not prove to be effective due the fact that the concentration was too low or that the type of HSL was not specific to this type of quorum sensing. There is also the possibility that HSLs act as a promoter of quorum sensing in this case rather than an inhibitor or a disruptor.

## 4. Conclusions

This research suggests that quorum sensing contributed to the interaction between *M. aeruginosa* and heterotrophic bacteria. The results revealed that heterotrophic bacteria can be grown in a system with *M. aeruginosa,* and the presence of HSLs can disrupt quorum sensing and prevent algal blooms. The development of *M. aeruginosa* blooms is favored by the interaction of multiple bacterial strains that are commonly present in freshwater samples, and the presence of other types of algal species could limit algal bloom if both algae compete for the same type of substrate. More importantly, environmental factors such as climate conditions and water quality parameters are not the only regulators of algal blooms. The interaction system is more complex than that and includes the presence of heterotrophic bacteria and quorum sensing.

The interruption of quorum sensing, therefore, blocking signal molecules with HSL is promising for the prevention of cases such as pet deaths which are reported on a regular basis in social media. Because dogs are sensitive to toxin exposition at lower levels, the future is not promising in terms of other sensitive individuals such as children and the elderly, who can be affected in a similar manner. As shown in this research, there are ways to explain the phenomena and alternative routes to prevent the effects of toxin production. However, the HSL used in this research did not prevent algal blooms. The only way to achieve such a prevention is a more drastic interference in which no nutrients should be added unnecessarily to water bodies. Warmer temperatures do contribute to algal bloom, but it is the food source that regulates its growth of bloom. Alternatively, future studies could also use biomass as commercially viable products or even in the energy sector. There is certainly a growing interest in studying cyanobacteria.

## Figures and Tables

**Table 1:** Gene identification, primer sequencing, and amplification conditions.

Gene	Primer Sequencing 5′ → 3′	Amplification Conditions
*16S*	8F: AGAGTTTGATCCTGGCTCAG 1492R: GGWTACCTTGTT ACG ACTT	One cycle at 94 °C for 5 min, followed by 35 cycles of 94 °C for 1 min, 55 °C for 1 min, and 72 °C for 2 min and elongation at 72 °C for 6 min.
*luxS*	luxSF: TCGGCGATGATGTGGTGATT luxSR: TTTTGTCGGGTGCGTGTTTG	One cycle at 94 °C for 5 min, followed by 35 cycles of 94 °C for 1 min, 55 °C for 1 min, and 72 °C for 2 min and elongation at 72 °C for 6 min.
*mcyB* ^1^	mcyBF: CCTACCGAGCGCTTGGG mcyBR:GAAAATCCCCTAAAGATTCCTGAGT	One cycle at 95°C for 3 min, followed by 40 cycles of 95 °C for 15 s, 59 °C for 30 s, and 72 °C for 30 s and elongation at 72 °C for 10 min.

^1^ Shao, Wu [37].

**Table 2:** Descriptive parameters and water quality analysis of untreated and treated samples of PCC7806, Cibolo Creek and Leon Creek, after 30 days of experiment and fresh water from day 1 of setting 1 (algal interaction) and setting 2 (heterotrophic interaction).

**Parameters (Setting 1)**	**Fresh Water**	**Untreated**			**Treated**		
		**PCC**	**CC**	**LC**	**PCC**	**CC**	**LC**
pH	7	9.7	9.8	9.7	9.8	9.8	9.4
Conductivity (μS/cm)	77.1	21.8	19.4	20.2	10.6	22.6	23
Alkalinity (mg/L)	360	240	240	240	240	240	240
Hardness (mg/L)	50	50	50	50	50	50	50
Nitrate (mg/L)	0	500	0	0	500	500	0
Nitrite (mg/L)	0	80	0	0	80	80	0
Chlorine (mg/L)	0	1	0.5	0.5	3	0.5	0
Total phosphorous (mg/L)	0.5	2	4.5	2.5	1.7	3.7	1.5
COD (mg/L)	40	162	160	160	162	149	152
Microcystin (μg/L)	0.5	1.3	1.9	0.7	1.4	1.4	1.4
**Parameters (Setting 2)**	**Fresh Water**	**Untreated**			**Treated**		
		**PCC**	**CC**	**LC**	**PCC**	**CC**	**LC**
pH	7	10.7	11.1	11.5	11	11	11.1
Conductivity (μS/cm)	77.1	13.6	74.8	77.8	13.7	75.6	76.3
Alkalinity (mg/L)	360	240	360	360	240	360	360
Hardness (mg/L)	50	25	50	50	25	50	50
Nitrate (mg/L)	0	0	0	0	0	10	10
Nitrite (mg/L)	0	0	1	1	0	1	1
Chlorine (mg/L)	0	0	0.5	0.5	0	0.5	0.5
Total phosphorous (mg/L)	0.5	1.8	2.3	1.7	1.8	2.7	2.8
COD (mg/L)	40	108	47	92	78	64	95
Microcystin (μg/L)	0.5	1.5	1.9	2.8	1.5	0.9	1.1

## Data Availability

The data presented in this study are available on request from the corresponding author. The data are not publicly available due to proprietary reasons at the time of the publication of this manuscript.
